# The Obese Brain: Mechanisms of Systemic and Local Inflammation, and Interventions to Reverse the Cognitive Deficit

**DOI:** 10.3389/fnint.2022.798995

**Published:** 2022-03-29

**Authors:** Verónica Salas-Venegas, Rosa Pamela Flores-Torres, Yesica María Rodríguez-Cortés, Diego Rodríguez-Retana, Ricardo Jair Ramírez-Carreto, Luis Edgar Concepción-Carrillo, Laura Josefina Pérez-Flores, Adriana Alarcón-Aguilar, Norma Edith López-Díazguerrero, Beatriz Gómez-González, Anahí Chavarría, Mina Konigsberg

**Affiliations:** ^1^Posgrado en Biología Experimental, Universidad Autónoma Metropolitana - Unidad Iztapalapa, Mexico City, Mexico; ^2^Departamento de Ciencias de la Salud, División de Ciencias Biológicas y de la Salud (DCBS), Universidad Autónoma Metropolitana Iztapalapa, CDMX, Mexico City, Mexico; ^3^Departamento de Biología de la Reproducción, DCBS, Universidad Autónoma Metropolitana Iztapalapa, Ciudad de México (CDMX), Mexico City, Mexico; ^4^Programa de Doctorado en Ciencias Biomédicas, Universidad Nacional Autónoma de México, CDMX, Mexico City, Mexico; ^5^Unidad de Investigación en Medicina Experimental, Facultad de Medicina, Universidad Nacional Autónoma de México, CDMX, Mexico City, Mexico

**Keywords:** obesity, cognitive decline, inflammation, oxidative stress, natural products, exercise, blood-brain barrier

## Abstract

Overweight and obesity are now considered a worldwide pandemic and a growing public health problem with severe economic and social consequences. Adipose tissue is an organ with neuroimmune-endocrine functions, which participates in homeostasis. So, adipocyte hypertrophy and hyperplasia induce a state of chronic inflammation that causes changes in the brain and induce neuroinflammation. Studies with obese animal models and obese patients have shown a relationship between diet and cognitive decline, especially working memory and learning deficiencies. Here we analyze how obesity-related peripheral inflammation can affect central nervous system physiology, generating neuroinflammation. Given that the blood-brain barrier is an interface between the periphery and the central nervous system, its altered physiology in obesity may mediate the consequences on various cognitive processes. Finally, several interventions, and the use of natural compounds and exercise to prevent the adverse effects of obesity in the brain are also discussed.

## Introduction

Obesity is a chronic and stigmatized disease that affects children, adolescents, adults, and elderly people (The Lancet Diabetes Endocrinology, 2017; Goisser et al., 2020; Smith et al., 2020; Bray and Ryan, 2021). Some authors do not consider obesity a disease but a state; the problem with this definition is that it has led to neglecting the importance of this sickness and serious measures have not been taken to counteract it. This has led to obesity becoming a growing public health problem with severe economic consequences (Hruby and Hu, 2015; Berthoud et al., 2020).

According to the World Health Organization (WHO), more than 1.9 trillion adults in the world are overweight, and 650 million are obese; besides, this prevalence has also dramatically increased in children and adolescents. Around 2.8 million people die each year because of this pandemic (World Health Organization [WHO], 2021).

Obesity arises due to an energy imbalance between calories consumed and calories expended, creating an excessive energy balance state that increases body weight. The WHO has defined obesity as an excessive accumulation of body fat mass that can affect health and is diagnosed in adults with a body mass index (BMI) ≥ 30 kg/m^2^ (Whitlock et al., 2009). However, the pathogenesis of obesity is much more complex than a simple imbalance between energy intake and expenditure that leads to the passive accumulation of excessive weight. The etiologies associated with obesity include diverse aspects, such as genetic and epigenetic factors, and psychological, social, and cultural features that make obesity and overweight a multifactorial disease (Heindel and Blumberg, 2019; Genario et al., 2020).

It is well known that obesity substantially increases the risk of metabolic and chronic diseases such as type 2 diabetes mellitus (DM2), some types of cancer, cardiovascular and musculoskeletal diseases (Castro et al., 2017; Blüher, 2019), along with other disorders such as depression and neurodegenerative diseases (Gainey et al., 2016; Buie et al., 2019). Interestingly obesity has been accepted as an important risk factor for cognitive impairment (Castanon et al., 2015; Dye et al., 2017). During obesity, adipose tissue produces cytokines such as IL1ß, IL6, IFNγ, TNFα, MCP1, promoting chronic inflammation (Guillemot-Legris and Muccioli, 2017). Chronic low-grade inflammation disrupts the blood-brain barrier (BBB) due to endothelial dysfunction, generating neuroinflammation and increasing oxidative stress, leading to cognitive decline (Tucsek et al., 2014; Castanon et al., 2015). It is essential to mention that, although there are few studies, obesity has been shown to impact men and women differently, so the outcomes related to cognitive decline, dementia, and other diseases might be different (Censin et al., 2019).

For all these reasons, in this review, we will focus on analyzing how obesity-related peripheral inflammation can affect the central nervous system (CNS), generating neuroinflammation. Since the BBB is the interface between the periphery and the central nervous system, it may be the link between peripheral inflammation and the consequences that obesity may have on various cognitive processes. Furthermore, since the WHO has outlined different measures to prevent the adverse effects of obesity, several of those interventions will be discussed.

## Obesity and Inflammation

### Changes in Adipose Tissue During Obesity

The adipose tissue is a specialized connective tissue classified into brown (BAT) and white adipose tissue (WAT) (Saely et al., 2012). BAT predominates in the newborn’s in the interscapular, perirenal, and inguinal regions. In adults, BAT is present in the neck, interscapular, and supraclavicular regions but is absent in the elderly and obese (Mittal, 2019). Sympathetic endings innervate BAT to mediate lipolysis since it specializes in heat generation by oxidating fatty acids through the dissipation of the proton gradient in the inner mitochondrial membrane. The generation of heat aims to thermoregulate body temperature (Wang et al., 2021).

Nowadays, WAT is considered an organ with relevant neuroimmune-endocrine functions, which participates in the organism’s homeostasis. One of the main functions of the WAT is the storage of fatty acids to provide these substrates to other tissues such as muscle during fasting or in periods of high energy demand. WAT also has a relevant role in appetite regulation, insulin resistance, cytokine secretion, mechanical protection, among others (Morigny et al., 2021). WAT is the most abundant adipose tissue distributed subcutaneously, perivascularly, and viscerally, the latter participating in metabolic dysregulations during obesity (Morigny et al., 2021; Porro et al., 2021). This adipose tissue is primarily composed of adipocytes, cells specialized in accumulating lipids. The WAT is also formed by the stromal cells, including pre-adipocytes, stem cells, endothelial cells, and immune cells such as macrophages, lymphocytes, and neutrophils (Ràfols, 2014). Macrophages and T lymphocytes have generated significant interest for their participation in the chronic low-grade inflammatory process present in obesity (Maurizi et al., 2018).

Macrophages can be classically (M1) or alternatively (M2) activated. Polarization into the M1 profile happens after pro-inflammatory tumor necrosis factor α (TNFα) and interferon γ (IFNγ) or toll-like receptor 4 (TLR4) signaling. In consequence, M1 macrophages express TNFα, interleukin (IL) 1α, IL1β, IL6, monocyte chemoattractant protein 1 (MCP1, also known as CCL2), chemokine (C-X-C motif) ligand 9 (CXCL9), and CXCL10, among other molecules. In turn, these factors attract unpolarized macrophages and induce the differentiation into the M1 state (Murray, 2017). Cytokines such as IL4 and IL13 induce M2 polarization. In turn, M2 macrophages secrete IL10, transforming growth factor β (TGFβ), chemokine (C-C motif) ligand 1 (CCL1), CCL17, CCL18, CCL22, and CCL24, favoring the differentiation of unpolarized macrophages into the M2 profile (Murray, 2017).

Under physiological conditions, the sensitivity toward insulin is maintained by releasing anti-inflammatory cytokines such as TGFβ and IL10 by the resident or M2 macrophages, favoring insulin-mediated glucose uptake (Li et al., 2020). Adipocytes release IL4 and IL13 (Table 1), promoting the polarization of macrophages to an M2 profile, thus favoring lipid metabolism and the secretion of TGFβ and IL10, ensuring a reduction in inflammation and resistance to insulin (Tsao et al., 2014; Akash et al., 2018).

**TABLE 1:** Main cytokines and their effects in obesity.

Cytokines	Cytokine source	Levels in obesity	Cytokine mechanisms in obesity	References
IL1β	• Subcutaneous adipose tissue • Visceral adipose tissue	↑ adipose tissue ↑ serum	• Induces Pre-adipocyte differentiation. • Reduction of insulin-induced glucose transport. • Inhibition of glucose uptake by adipocytes via ERK signaling. • Acts synergistically with TNFα and IL6, altering the lipase activity, leading to lipid accumulation in the liver and muscle. • Contribution to hepatic lipogenesis, triglyceride accumulation, and development of hepatic steatosis. • IL6 production. • T cell and macrophage activation.	Jager et al., 2007; Um et al., 2011; McArdle et al., 2013; Negrin et al., 2014; Wang et al., 2021
IL2	• Visceral adipose tissue • CD4 + and CD8 + T cells • Dendritic cells • Macrophages	↑ adipose tissue ↑ serum	• T cell activation. • Induction of inflammatory molecules like IL8, IL12A, CCL5, CCL19, CCR2, and CCR5. • Contribution to increased insulin resistance secondary to TLR2, TLR4, and TLR10 interaction.	Liu and Nikolajczyk, 2019; Kochumon et al., 2020
IL4	• TH2 cells • Visceral adipose tissue • M2 macrophages	↓adipose tissue ↓serum	• Inhibits lipid deposits. • Inhibits adipogenesis through the expression of peroxisome proliferator-activated receptor γ (PPARγ). • Promotes lipolysis due to binding to hormone-sensitive lipase (HSL).	Tsao et al., 2014; Lu et al., 2015; Shiau et al., 2019
IL6	• Subcutaneous adipose tissue • Visceral adipose tissue • Monocytes • M1 macrophages	↑ adipose tissue ↓ hypothalamus	• Promotes energy consumption by stimulating the hypothalamus. • Correlation with high TNFα levels and insulin resistance. • Chemotaxis and monocyte infiltration in adipose tissue by the expression of CD11b and CD163.	Sindhu et al., 2015; Kern et al., 2019; El-Mikkawy et al., 2020; Wang et al., 2021
IL10	• TH2 cells • Regulatory T cells • B cells • M2 macrophages	↓ adipose tissue ↓ serum	• Inhibition of pro-inflammatory cytokine synthesis by suppressing NF-kB in macrophages. • Association with hypertriglyceridemia by the affection of the JAK-STAT 3 signaling pathway.	Azizian et al., 2016; Kondo et al., 2018; Liu et al., 2018
IL13	• TH2 cells	↑ serum	• Polarization of macrophages into an M2 profile through the IL-13Rα1/IL-4R receptor. • Decrease insulin resistance. • Involved in increasing inflammation via the NLRP3 inflammasome. • Increases fatty acid oxidation in muscle.	Duffen et al., 2018; Martínez-Reyes et al., 2018; Knudsen et al., 2020
IL17	• Th17 cells in visceral adipose tissue • M1 macrophages • Neutrophils	↑ adipose tissue	• Inhibition of adipocyte differentiation. • Increase of inflammatory molecules like COX_2_ and PEG_2_. • Induction of IL6 synthesis by adipocytes. • CDK5-dependent phosphorylation of PPARγ in adipocytes, favoring gene expression related to diabetes.	Ahmed and Gaffen, 2010; Liu and Nikolajczyk, 2019; Teijeiro et al., 2021
IFNγ	• TH1 cells	↑ adipose tissue ↑ serum	• Macrophage regulation switching to the M1 profile. • Increase of insulin resistance. • Increase of adipocyte cell size.	Wada et al., 2011; O’Rourke et al., 2012; Wang et al., 2014; Surendar et al., 2019
MCP1 (CCL2)	• M1 macrophages	↑ adipose tissue ↑ serum	• Participation in adipogenesis promoting adipocyte growth. • Facilitation of insulin resistance and glucose intolerance. • Recruitment of immune cells.	Rocha et al., 2008; Cranford et al., 2016
TGFβ	• Regulatory T cells • M2 macrophages • Platelets	↑ serum	• Increase insulin resistance through TGFβ/Smad3 signaling via the repression of the insulin promoter and suppression of insulin level and secretion. • Inhibition of adipocyte differentiation. • Correlation with high levels of serum glucose.	Yadav et al., 2011; Zamani and Brown, 2011; Hong et al., 2016; Lee, 2018
TNFα	• TH1 cells • Subcutaneous adipose tissue • Visceral adipose tissue • M1 macrophages	↑ adipose tissue ↑ serum	• Inhibition of GLUT4 membrane translocation. • Induction of the serine phosphorylation of insulin substrate-1, leading to insulin resistance. • Suppression of the lipoprotein lipase activity. • Inhibitor of adipocyte differentiation. • Suppression of genes involved in uptake and storage of non-esterified fatty acids and glucose.	Bennet et al., 2006; Tzanavari et al., 2010; Kern et al., 2019; Liu and Nikolajczyk, 2019; Alzamil, 2020; Wang et al., 2021

During obesity, excessive energy coming from the diet and the lack of physical activity promotes lipid storage in adipocytes (Trayhurn, 2013; Maurizi et al., 2018). If that persists, adipocytes broaden in a phenomenon called hypertrophy. The pre-adipocyte differentiation into adipocytes complements this process to compensate for the growth of existing ones to maintain a balance in the storage capacity, in a phenomenon called hyperplasia (Choe et al., 2016).

Because of cytokine release, especially TNFα, a high number of classically activated M1 macrophages infiltrate the adipose tissue, which is associated with insulin resistance (Akash et al., 2018). Those changes alter the WAT microenvironment, favoring the expression of pro-inflammatory adipokines and the activation of adipose tissue macrophages (ATMs) to a pro-inflammatory or M1 profile, promoting the secretion of more pro-inflammatory molecules, such as TNFα, IL1β, IL6, and MCP1 (Table 1), generating an increased accumulation of ATMs, which in turn secrete a higher amount of pro-inflammatory cytokines (Sam and Mazzone, 2014). A marker of damage caused by ATMs infiltration is the formation of crown-like structures characterized by M1 ATMs expressing CD11c around dead adipocytes. These structures are increased in obesity and are related to inflammation and insulin resistance (Lumeng et al., 2007; Sam and Mazzone, 2014).

In normal conditions, WAT is highly irrigated, ensuring adequate transport of nutrients and oxygen from the diet; nevertheless, in obesity, adipocyte hypertrophy hinders the diffusion of oxygen and nutrients, causing a hypoxic state (Sam and Mazzone, 2014). The cellular environment with low oxygen tension elicits hypoxia-sensitive genes that activate major hypoxia-inducible molecules (HIF) and inflammatory transcription factors such as NF-κB (Trayhurn, 2014), triggering a change in the adipokine secretion profile to enter into cellular stress, both at the mitochondria and the endoplasmic reticulum (Deng and Scherer, 2010). This first change alters the local microenvironment, as pro-inflammatory cytokines increase and the ATMs change to an M1 phenotype producing TNFα, IL1β, IL6, and MCP1, culminating in a local inflammatory process and a significant number of infiltrating macrophages (Tsao et al., 2014). In obese mice, the lack of oxygen in WAT generates changes related to the dysfunction of adipocytes, where it is worth highlighting the increase in adipokines related to inflammation (IL6, leptin, Angptl4, and VEGF), in addition to an increase in lactate production and the induction of fibrosis and insulin resistance (de Oliveira and Mafra, 2013; Trayhurn, 2014). Also, elevated adipose HIF1A protein and RNA levels are present in patients with obesity class 3, confirming hypoxia in WAT (Todorćević et al., 2021).

Among the various bioactive molecules produced by adipocytes are adipokines such as TNFα, leptin, resistin, and plasminogen activator inhibitor type 1 (PAI-1) (Deng and Scherer, 2010). The loss of their regulation during obesity is related to the pathophysiology of metabolic diseases; for example, decreased adiponectin levels are associated with DM2 (Frankenberg et al., 2017). Its main functions include glucose and lipid metabolism, and the prevention of inflammation. The mechanism through which it exerts these functions has not been explicitly explained.

### Inflammatory Mediators in Obesity

As mentioned before, the alteration of adipocytes causes ATMs to polarize to an M1 profile, synthesizing and secreting pro-inflammatory cytokines, which are considered obesity inflammatory mediators and have diverse effects (Sam and Mazzone, 2014). Ym1, arginase 1, and IL10 gene expression is observed in lean mice, stimulating an M2 activation on ATMs as an anti-inflammatory mechanism. While in obese mice, the transcription of TNFα and iNOS genes increase, contributing to TNFα-induced insulin resistance (Lumeng et al., 2007; Sam and Mazzone, 2014).

Among the mentioned cytokines, IL6 regulates multiple aspects of metabolism like the regulation of adipose tissue, lipolysis, oxidative metabolism, and energy expenditure (Wueest and Konrad, 2020). Adipose tissue, endothelial cells (vascular stroma), fibroblasts, macrophages, monocytes, and lymphocytes secrete IL6 contributing to acute phase reactions, chronic inflammatory processes, and homeostatic energy regulation, influencing obesity and insulin resistance (Stȩpień et al., 2014; Han et al., 2020). During obesity, adipose tissue increases leptin secretion and suppresses satiety, promoting gluconeogenesis and hepatic insulin resistance (Han et al., 2020). Furthermore, one-third of total IL6 circulating levels is produced in adipose tissue (Makki et al., 2013).

Tumor Necrosis Factor α is a peptide secreted by different cells types like monocytes, macrophages, and microglia. In adipose tissue, pre-adipocytes, stromal vascular cells, and infiltrating macrophages also secrete TNFα, where it suppresses the genes involved with the internalization and storage of non-esterified fatty acids, glucose, and transcription factors involved in adipogenesis and lipogenesis (Tzanavari et al., 2010). MCP1 is a chemokine produced by adipocytes and M1 macrophages; it recruits monocytes/macrophages for their infiltration into adipose tissue, and its concentration increases in response to IL1, TNFα, and TLR4 signaling (Surmi and Hasty, 2008; Rajasekaran et al., 2019). MCP1 increases lipolysis and leptin secretion while lowering insulin-stimulated glucose uptake, increasing plasma levels during obesity, contributing to impaired insulin sensitivity (Makki et al., 2013).

Interleukin 1 is a family of cytokines in which IL1α and IL1ß stand out. These cytokines are crucial in innate inflammatory responses, being responsible for fever; however, in recent decades, they have been recognized for their participation in the progression of insulin resistance induced by obesity due to their elevated plasma levels and increased inflammasome NLRP3 activity (Di Renzo et al., 2007; Ballak et al., 2015).

Leptin, the product of the *LEP* gene, is a 16 kDa peptide hormone secreted mainly by adipose tissue (Pérez-Pérez et al., 2020). Its main function is the homeostatic regulation of appetite and body weight through the induction of anorectic factors and the expression of orexigenic neuropeptides in the hypothalamus (Vera et al., 2018). Circulating leptin levels correlate with body weight. Therefore, obese people tend to produce more leptin than slimmer people. Mice and humans with leptin deficiency attain intense hyperphagia and develop severe obesity and various metabolic and endocrine disorders (Paz-Filho et al., 2012). Leptin may participate in the activation and maintenance of the inflammatory response due to its ability to regulate innate and adaptive immune responses (Bernotiene et al., 2006). In innate immunity, leptin increases the cytotoxicity of natural killer (NK) cells, and induces the activation of a wide range of cells such as granulocytes, dendritic cells and macrophages. In the adaptive immune response, leptin increases the proliferation of naive T lymphocytes and B lymphocytes and decreases regulatory T lymphocytes (Treg). Leptin can polarize T helper cells (Th) toward a pro-inflammatory (Th1) rather than an anti-inflammatory (Th2) phenotype (Abella et al., 2017). In addition, pro-inflammatory cytokines increase leptin synthesis and release, which perpetuates the chronic inflammatory state characteristic of obesity.

Another mediator of inflammation during obesity is the oxidative stress (OS) generated in fat tissue. OS is defined as the imbalance between the oxidant molecules generated by the cells and the antioxidant systems that neutralize them (Thannickal and Fanburg, 2000). It is well recognized that OS and inflammation are damaging events that enhance each other. During obesity, the pro-inflammatory adipokines activate signaling cascades that can stimulate enzymes that generate reactive oxygen species (ROS), such as NADPH oxidase (NOX), which mainly produce superoxide radicals and hydrogen peroxide (Hsu et al., 2019). ROS, especially the hydroxyl radical, can oxidize proteins, damage membrane lipids and DNA, increasing the risk of degenerative diseases. In the CNS, the nitric oxide synthase (NOS) is also activated, generating nitric oxide, which produces the peroxynitrite anion that nitrates proteins, damaging them. Increased NOS activity is associated with increased calcium and excitotoxicity (Brown, 2010; Yuste et al., 2015). Inflammation has been related to mitochondrial dysfunction that causes a decrease in ATP levels and an increased ROS generation, thus enhancing OS. The changes toward a more oxidized state activate the NLRP3 inflammasome and transcription factors such as NF-κB, which in turn induce the synthesis of more pro-inflammatory cytokines that activate immune cells, thus perpetuating the damage of the OS and inflammation (Morgan and Liu, 2011; Sandhir et al., 2017). The chronic low-grade inflammation produced by adipocytes generates OS and creating a vicious cycle that alters the functions of the immune, endocrine, and nervous systems and has been associated with the establishment of metabolic, cardiovascular, and degenerative diseases.

**FIGURE 1:**
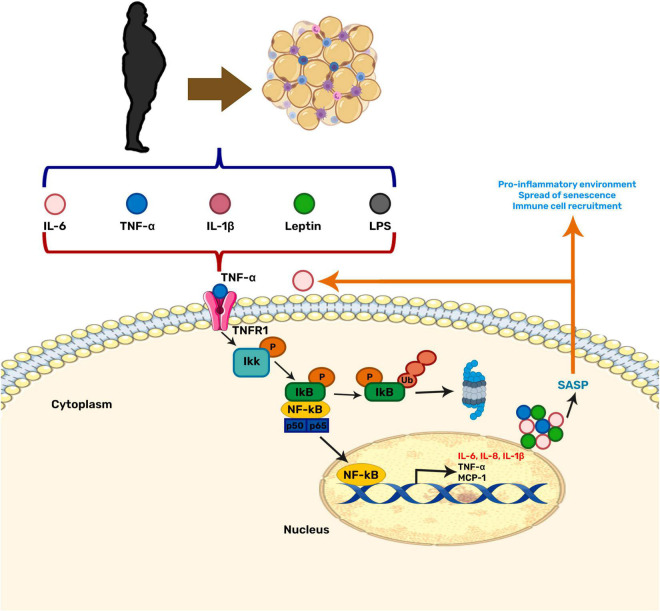
Relationship between obesity-related inflammation and senescence. NF-κB is the central regulator in the stress response and may be activated by various stimuli. Among them, pro-inflammatory cytokines secreted by adipose tissue during obesity. This factor has also been related to the aging process by contributing to cellular senescence through the senescence-associated secretory phenotype (SASP).

Moreover, inflammation and OS can induce cellular senescence (Burton and Faragher, 2018). Senescence is a stress response state in which cells lose their ability to proliferate and secrete a set of pro-inflammatory and growth factors, proteases, among other proteins, known as the senescence-associated secretory phenotype (SASP). The SASP attracts immune cells aiming to remove damaged cells, hence promoting the restoration of cell homeostasis (Burton and Faragher, 2018). Senescent cells can be beneficial when they contribute to tumor suppression, but, in the long term, they promote tissue deterioration during aging. During obesity, the inflammation and OS generated in the brain increases the amount and accumulation of senescent cells, thus contributing to the neuroinflammation due to the SASP secretion. The neuroinflammation induced by the senescent cells creates a vicious cycle that escalates inflammation and OS, and has been linked to age-related diseases (Maciel-Barón et al., 2017; Ogrodnik et al., 2019; Figure 1).

High-fat diets (HFD) in experimental rodent models cause OS, increased circulating pro-inflammatory cytokines, and the appearance of senescent markers (Minamino et al., 2009; Cavaliere et al., 2018). The KK-A*^y^* mouse model with ectopic expression of the Agouti-related protein (AgRP) is hyperphagic and develops severe obesity. These animals increased ROS generation though they were fed with a standard diet. The KK-A*^y^* mice adipose tissue exhibited a senescent phenotype, characterized by enhanced β-galactosidase activity, high p53 protein, and elevated expression of CDK1 mRNA compared to wild-type mice. Increased expression of the pro-inflammatory cytokines TNFα and MCP1, and macrophage markers in the adipose tissue of KK-A*^y^* mice were also observed, suggesting that excessive calorie intake may induce senescence-like changes in adipose tissue (Minamino et al., 2009).

## Chronic Obesity and Neuroinflammation

Systemic inflammation, particularly low grade chronic inflammation, such as the one generated during obesity, has been reported to cause changes in the brain and induce neuroinflammation (Ellulu et al., 2017; Ugalde-Muñiz et al., 2020). The neuroinflammatory response during obesity occurs in different structures of the CNS, such as the cerebellum, amygdala, cerebral cortex, and hypothalamus (Guillemot-Legris and Muccioli, 2017; Jais and Brüning, 2017; Van Dyken and Lacoste, 2018). Of particular interest in the context of obesity-induced cognitive decline is the neuroinflammation generated in the hippocampus, since experimental animal models subjected to diets rich in fat and carbohydrates have shown learning and memory deficits. This neuroinflammation has also been associated with changes in the integrity of the blood-brain barrier (BBB), so these structures will be discussed next (Bruce-Keller et al., 2009).

### Blood-Brain Barrier as an Interface Between the Periphery and the Central Nervous System

**FIGURE 2:**
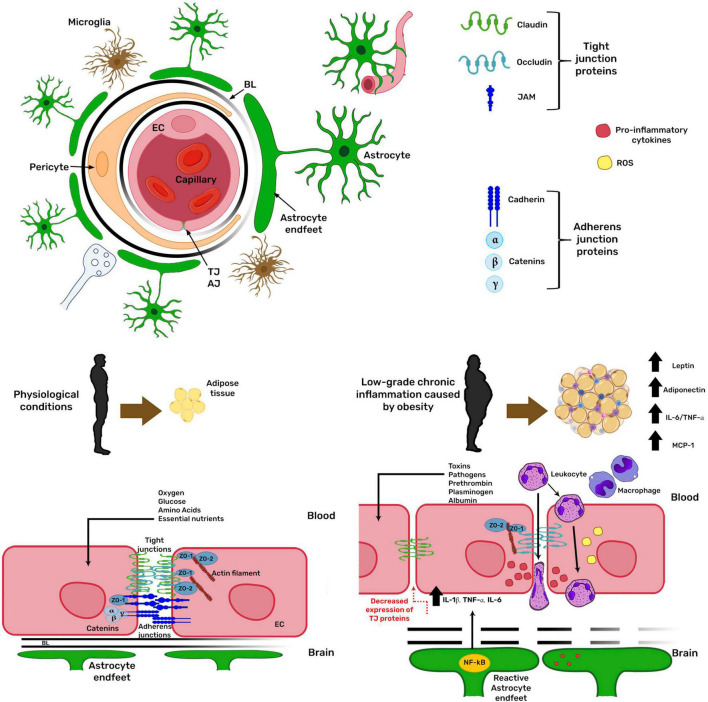
Obesity modifies blood-brain barrier physiology. The blood-brain barrier (BBB) is formed by brain endothelial cells (EC), which acquire its barrier phenotype by their cellular interactions with pericytes and the soluble factors released by astroglia. The barrier restricts the unregulated diffusion of macromolecules between blood and the brain. In obesity, low-grade chronic inflammation increases BBB permeability. Obesity-related inflammation depends on the NF-κB pathway, increasing the expression of pro-inflammatory proteins, related to decreased tight junction protein expression, deranging BBB integrity. AJ, adherens junction; TJ, tight junction; BL, basal lamina; ZO, zonula occludens; IL, interleukin; TNFα, tumor necrosis factor α; MCP1, monocyte chemoattractant protein 1.

The communication between the peripheral tissues capable of acquiring, detecting, and storing nutrients with the specialized nuclei in the CNS is essential to regulate nutrition and metabolism. The BBB is the interface for these communications because several signals are transferred through the blood (Rhea et al., 2017). The BBB is located at 99% of brain capillaries (Pachter et al., 2003) and is formed by brain endothelial cells, which acquire its barrier phenotype by their cellular interactions with mural cells, such as pericytes, and by the soluble factors released by the astroglia (Daneman and Prat, 2015). The BBB ensures that the composition of the interstitial fluid is adequate for the physiological properties of each brain region (Fletcher and Callanan, 2012). It protects the brain from toxic substances present in the circulation, conferring a chemical and physical barrier to the CNS (Fletcher and Callanan, 2012) as it restricts the unregulated diffusion of macromolecules between the blood and the CNS and selectively regulates the transport of circulating nutrients and hormonal signals from the blood to the brain and *vice versa* (Rhea et al., 2017). The BBB structure involves the establishment of inter-endothelial tight junctions and the expression of specialized carrier systems. Inter-endothelial tight junctions are formed by the transmembrane proteins claudin, occludin, and junctional adhesion molecule (JAM), which are anchored to the cytoskeleton through their interaction with adaptor proteins of the MAGUK family, such as zonula occludens-1 (ZO-1) and ZO-2 (Daneman and Prat, 2015; Figure 2). Another junction type at the BBB is the adherens junction, formed mainly by the transmembrane protein cadherin docked to the cytoskeleton through catenins (α, β, and γ). Adherens junctions are a prerequisite for tight junction assembly and maintenance (Kadry et al., 2020). When the BBB function is lost, potentially neurotoxic molecules within the bloodstream, such as prothrombin, plasminogen, and albumin, can freely enter the brain (Fletcher and Callanan, 2012). The inflammation caused by obesity has been related to changes in BBB permeability (reviewed in Hurtado-Alvarado et al., 2016), inducing leukocyte extravasation along with the potential entry of pathogens and toxins into the CNS, which in turn stimulate more inflammatory responses, causing a vicious cycle (Van Dyken and Lacoste, 2018). All these mechanisms are regulated by the NF-κB pathway increasing the expression of pro-inflammatory proteins such as IL1β, TNFα, and IL6. The increase in pro-inflammatory cytokines is related to the decrease in tight junction protein expression and disturbed BBB integrity (Van Dyken and Lacoste, 2018; Figure 2).

### The Role of Diet and Aging in the Blood-Brain Barrier Function

As mentioned above, changes in the BBB structure and function during obesity may cause further pathologies in the CNS, increasing neuroinflammation and cognitive decline (Rhea et al., 2017). Astrocytes and microglia are essential in maintaining the BBB integrity supporting neuronal metabolism, and preventing/responding to local tissue injury, and both cell types are activated in the brain of rodents and humans with HFD consumption (Rhea et al., 2017).

A high fat and glucose diet administrated for 90 days to juvenile male Sprague Dawley rats increased BBB permeability to sodium-fluorescein, a low molecular weight exogenous tracer, in the hippocampus. This effect was related to a lower expression in the mRNA of the tight junction proteins claudin-5, claudin-12, and occludin, and deficits in hippocampal-related learning and memory (Kanoski et al., 2010). Likely, a Mediterranean diet, rich in saturated fat and dextrose, administrated to male Sprague Dawley rats for 10, 40, and 90 days increased the BBB permeability to sodium-fluorescein at day 90 in restricted regions of the hippocampus and the dorsal striatum; indicating that the loss of barrier function is gradual. This study also associated the increase in the BBB permeability with the deficits in the hippocampus-dependent learning and memory (Hargrave et al., 2016).

Another diet rich in cholesterol administered to male C57 BL/6 mice during ten weeks potentiated the effect of ischemia on BBB permeability by increasing the extravasation of immunoglobulin (IgG) in the frontal cortex as compared to ischemic mice fed with a standard diet (ElAli et al., 2011). Another study showed that obesity at old age increases cognitive decline, particularly in the hippocampus. Young (7 months) and old (24 months) male C57 BL/6 mice received either a standard diet or a HFD. Cognitive impairment in obese and aged mice was associated with decreased microvascular density and pericyte coverage in the hippocampus and cerebral cortex; in addition, reduced blood flow in the cerebral cortex was related to memory problems (Tucsek et al., 2014).

Under pathological conditions, such as DM2, increased BBB permeability has also been reported. A rodent model of streptozotocin-induced DM2 increased BBB permeability to low-molecular-weight tracers earlier in the midbrain (at 28 days post-induction) and later in the hippocampus, basal nuclei, and cerebral cortex (at 56- and 90-days post-induction). However, for large molecules (e.g., Evans blue), increased BBB permeability in diabetic animals was observed until later times (more than 56 days post-induction) and only in the midbrain and basal nuclei (Huber et al., 2006).

### Glial Activation in Obesity

This loss of BBB permeability during the chronic low-grade inflammatory state associated with obesity facilitates that pro-inflammatory molecules access to the brain parenchyma, thus allowing them to interact with the microglia (Ouyang et al., 2014; Gianfrancesco et al., 2019; González-Olmo et al., 2021). Furthermore, it has been reported that there is increased activation and proliferation of microglia and astrocytes in both obese humans and rodent models of obesity (Castanon et al., 2015).

**FIGURE 3:**
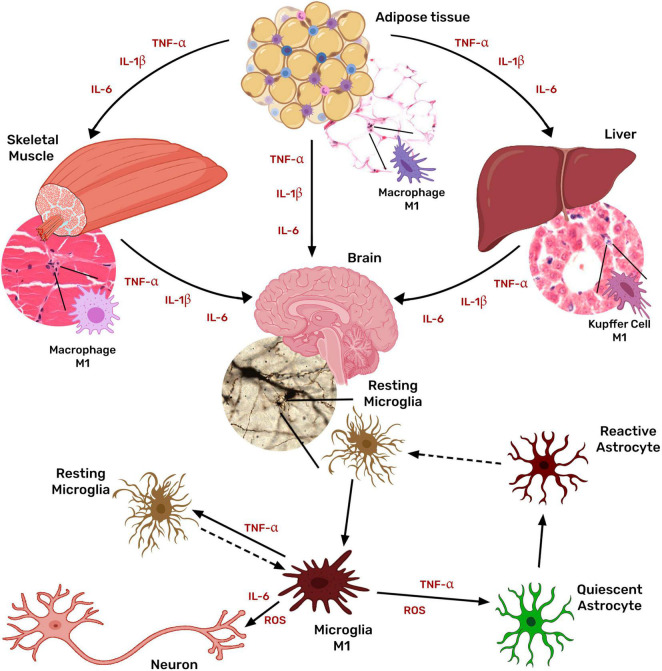
Systemic inflammation associated with obesity activates microglia and astrocytes. Obesity chronic low-grade inflammation is mediated mainly by tissue macrophages in the adipose tissue through the secretion of TNF, IL1β, IL6, and MCP1. Cytokines originated in the adipose tissue stimulate macrophages of other tissues (liver, muscle, and even brain) further to produce TNF, IL1β, IL6, and MCP1, inducing a generalized inflammatory state. Peripheral inflammatory signals also activate microglia, which then secretes more inflammatory cytokines, activating astrocytes and favoring a chronic neuroinflammatory condition that leads to neuronal damage.

Microglia, the brain-resident macrophage, responds to peripheral inflammatory signals by its activation and thus the secretion of more inflammatory cytokines, perpetuating the neuroinflammatory condition and leading to neuronal damage (Thaler et al., 2012; Figure 3).

After stimulation, the microglial cells are activated to a state of “priming or pre-activation” (Perry and Holmes, 2014). This state makes the microglia more sensitive to pro-inflammatory stimuli. Microglial priming occurs during aging, neurodegenerative diseases, and traumatic brain injury (Perry and Holmes, 2014; Li J. W. et al., 2018). However, evidence has begun to emerge that systemic inflammation, either induced with peripheral LPS or the administration of one or more of the pro-inflammatory cytokines such as TNFα, IL1β, IL6, IL33, is involved in microglial priming (Perry and Holmes, 2014; Li J. W. et al., 2018). Due to the heterogeneity of microglial density depending on the brain region, brain structures are differentially affected; the main affected brain regions are the hypothalamus, hippocampus, cerebral cortex, and striatum (Milanova et al., 2021). Another example is the NLRP3 protein of the inflammasome in visceral adipose tissue, which directly affects IL1ß levels in the brain, activating microglia through the IL1R1 receptor and thus affecting memory in obese animals (Guo et al., 2020).

Microglial cells rapidly react to HFD, inducing morphological changes in the hypothalamus (Thaler et al., 2012). HFD increases Iba-1 expression in the arcuate nucleus (ARC), paraventricular nucleus (PVN), and hippocampus. No specific microglial changes are observed in the cerebral cortex and striatum (Thaler et al., 2012; Milanova et al., 2021). In the medio -basal hypothalamus (MBH), microgliosis is mediated by activating pathways such as NF-κB, favoring cell infiltration, increased food intake, and local inflammation (Valdearcos et al., 2017). As mentioned above, the activation of these inflammatory pathways is crucial because the activation of IKKβ/NF-κB influences leptin and insulin metabolism, affecting even the processes of glucose intolerance in obesity (Zhang et al., 2005).

The hypothalamic microglia has been one of the most studied for its importance in metabolic regulation; this microglia acts as a sensor that regulates the function of the hypothalamus and is very sensitive to changes; for example, males are more susceptible to neuroinflammation in this brain area (Dorfman et al., 2017; Rosin and Kurrasch, 2019). CX3CL1-CX3CR1 signaling seems to be relevant in microglial regulation, metabolic homeostasis, and obesity susceptibility. Male mice fed with HFD presented lower expression of CX3CL1-CX3CR1 in the hypothalamus, while HFD-female preserved both ligand and receptor normal expression (Dorfman et al., 2017).

In the hypothalamus, microglial activation is related to alterations in the organelles responsible for energy metabolism, i.e., the mitochondria. HFD increases the mitochondrial number and the mRNA expression of the uncoupling protein 2 (UCP2); the selective deletion of UCP2 in microglia prevents diet-induced obesity (Kim et al., 2019).

However, microglia cells are not the only component in neuroinflammation secondary to obesity; other glia, specifically astrocytes, have been extensively studied in an inflammatory setting. The glial-vascular mechanism in which astrocytes and endothelial cells are involved modulates microglial activation and, therefore, inflammation. In *in vitro* experiments, the activation of the endothelium favored microglia differentiation into the amoeboid forms and increased the release of TNFα, IL1β, and IL10, while IGF1 levels decreased. In contrast, microglia exposed to conditioned medium from activated astrocytes showed a M2 phenotype and higher levels of IGF1 secretion; a promotion of phagocytosis was also observed (Xing et al., 2018).

Multiple studies show astrocyte importance in obesity. Hypothalamic astrocytes accumulate lipid droplets in an obese environment, favoring astrogliosis and inflammatory markers such as TNFα, IL1β, IL6, MCP1, stimulating microglia, and other astrocytes, enhancing the inflammatory response (Kwon et al., 2017).

The selective isolation of microglia and astrocytes has made it possible to differentiate and identify the molecules involved in neuroinflammation and their changes over time. In a HFD animal model, on day 3 of diet administration, the microglia TNFα expression was elevated while astrocytic IL10 increased; after 28 days of the diet, both astrocytes and microglia became clearly inflammatory with high expression levels of TNFα (Sugiyama et al., 2020).

Microglial activation also correlates with deficits in hippocampal function in obesity models. Hippocampal dysfunction was secondary to increased synaptic phagocytosis and neuronal elimination after 3 months of HFD; regular diet reversed this effect and normalized hippocampal function (Hao et al., 2016). Likely, blockade of specific microglial receptors, such as fractalkine-receptor, prevents the loss of dendritic spines and cognitive decline in obese mice (Cope et al., 2018).

### Inflammatory Molecules in the Obese Brain

The inflammatory molecules in the obese brain have been studied extensively by testing different diets in animal models. For example, high-sugar diets, which promote cognitive decline in young animals, are associated with high IL6 and IL1β levels in the dorsal hippocampus (Hsu et al., 2015). The cafeteria diet model increased Iba-1 expression in the obese brain (de Oliveira et al., 2021). Chronic HFD feeding for 12 weeks enhanced TNFα, IL6, and leptin levels in the hippocampus and also promoted microglial activation in the prefrontal cortex and hippocampus (Gomes et al., 2020).

Dietary changes may reverse or protect against obesity-induced neuroinflammation. Modifying the Western diet, high in fat and low in fiber, decreased cognitive deterioration by adding β-glucans, prominent soluble fibers. Also, obese animals treated with β-glucans diminished microglial activation, TNFα, IL1β, and IL6, favoring hippocampal synaptogenesis markers (Shi et al., 2020).

In the obese brain, multiple pathways and molecules are affected (Table 2). TNFα is a crucial cytokine in neuroinflammation secondary to obesity, even critical in glucose metabolism by attenuating insulin signaling pathways and increasing levels of IL6, activating a neuroinflammatory state (Clemenzi et al., 2019). In addition, TNFα is associated with anxiety secondary to obesity since its pharmacological blockade improves anxiolytic triggers in obese animals (Fourrier et al., 2019). Labban et al. (2020) fed rats with HFD for 4 weeks and found an increase in pro-inflammatory cytokines, such as IL6 and IL12 in serum, an increase in brain OS markers, a decrease in brain serotonin levels, and an increase in brain dopamine and glutamate levels.

**TABLE 2:** Main cytokines in the CNS during obesity and their effects in neuroinflammation.

Cytokines	Cytokine source in the CNS	Expression in CNS cells in obesity	Cytokine mechanisms in CNS in obesity	References
IL1β	• Microglia • Neurons • Astrocytes • Oligodendrocytes	↑ Astrocytes ↑ Microglia	• Leukocyte recruitment to the CNS. • Rapid cellular infiltration to the brain parenchyma. • Increased MCP1(CCL2) expression by astrocytes and ICAM1 on vascular endothelial cells. • Impairment of hippocampal-dependent memory processing. • Regulation of food intake. • Increased neuronal cell death.	Shaftel et al., 2008; Dorfman et al., 2017; Ding et al., 2018; Lainez and Coss, 2019; Guo et al., 2020
IL2	• Neurons	↓ Neurons	• T cell proliferation. • Inhibition of the development of Th17 cells. • Reduction of neutrophil infiltration. • Diminishment of tight junction proteins degradation. • Expression of CD206.	Hoyer et al., 2008; Gao et al., 2017
IL4	• M2 microglia • TH2 cells	↓ Neurons	• M2 microglial phenotype differentiation. • Increased microgliosis and astrogliosis. • Expression of CD206. • Decreased production of inflammatory cytokines such as TNFα.	Gadani et al., 2012; Luzina et al., 2012; Latta et al., 2015; Rossi et al., 2018; Daseke et al., 2020
IL6	• Microglia • Astrocytes • Neurons • Endotelial cells	↑ M1 microglia ↑ Astrocytes ↑ Neurons	• Differentiation of oligodendrocytes. • Modulation of microglial activation. • Induction of nerve injury. • Bodyweight loss induced by enhanced leptin signaling through the STAT-3 pathway.	Szelényi, 2001; Thaler et al., 2012; Le Foll et al., 2015; Rothaug et al., 2016; Bobbo et al., 2019; Hu et al., 2020; Recasens et al., 2021
IL10	• Regulatory T cells • B cells • Neurons • Microglia • Epithelial cells	↓ Neurons ↓ Microglia	• Vascular remodeling. • Reduction of leukocyte adhesion and extravasation. • Regulation of the NFκB signaling. • Improvement of neurogenesis.	Pereira et al., 2015; Azizian et al., 2016; Garcia et al., 2017; Kondo et al., 2018; Liu et al., 2018
IL17	• Th17 cells • T CD4 + cells • T CD8 + cells	↑ Th17 cells ↑ T CD4 + cells ↑ T CD8 + cells	• Induction of the NFκB pathway. • Contribution to the BBB permeability. • M1 polarization of microglia. • Activation of glial cells to produce inflammatory mediators, matrix metalloproteinases, chemokines, and free radicals.	Basu et al., 2015; Yang and Yuan, 2018; Qiu et al., 2021; Chen et al., 2022
TGFβ	• Regulatory T cells • Oligodendrocytes • M2 Microglia • Astrocytes	↑ Astrocytes	• Free radical production through NOX1. • Cytotoxicity and neurodegenerative changes through the SMAD3 pathway. • Expression of inflammatory genes in pericytes like NOX4, COX2, IL6, and MMP2.	Von Bernhardi et al., 2015; Rustenhoven et al., 2016; Patel et al., 2017
TNFα	• Astrocytes • M1 Microglia	↑ Astrocytes ↑ M1 Microglia	• Increase of the anorexigenic POMC activity. • Potentiation of glutamate-mediated cytotoxicity. • Induction of the NFκB pathway and secretion of IL1β. • Affection of the spatial learning and memory function. • M1 phenotype polarization.	Belarbi et al., 2012; Thaler et al., 2012; Olmos and Lladó, 2014; Lainez and Coss, 2019; Rodrigues et al., 2020

*BBB, blood brain barrier; CNS, central nervous system; COX2, Cyclooxygenase 2; ICAM1, intercellular adhesion molecule,1; MCP1, monocyte chemoattractant protein 1; MMP2, matrix metalloproteinase 2; NADPH, nicotinamide adenine dinucleotide phosphate; NFκB, Nuclear factor kappa B; NOX1, NADPH oxidase 1; NOX4, NADPH Oxidase 4; POMC, proopiomelanocortin.*

### Consequences of Obesity: Cognitive Decline

The obesity-related neuroinflammation, the BBB integrity loss, and the microglial activation induce synaptic remodeling, neuronal apoptosis, and decreases neurogenesis, which have been associated with cognitive decline (Miller and Spencer, 2014; Li J. W. et al., 2018; Zhou et al., 2020).

Studies performed with obese animal models have shown a relationship between diet and cognition (Castanon et al., 2015; Duffy et al., 2019). Rodents fed with HFD have shown deficiencies in working memory and learning (Nguyen et al., 2014; Gainey et al., 2016). Moreover, OS and inflammation promoted by obesity contribute to neuronal damage and cognitive failure. Zhang et al. (2005) reported that HFD administration to male Sprague-Dawley rats for 5 months activated NF-κB pathway, increased ROS production and NOX expression in the cerebral cortex, as well as prostaglandin E2 (PGE2), cyclooxygenase 1 (COX1), and COX2 levels, contributing to neuronal damage and cognitive deterioration. It has been reported that feeding C57B1/6 mice with HFD for 16 weeks modified the redox state and decreased Nrf2 activation, contributing to cognitive impairment evaluated by 14-Unit Stone Maze (Morrison et al., 2010). Something similar was observed in Wistar rats fed a high-calorie diet (HCD) for 13 weeks; these rats presented memory loss evaluated with the Morris water maze, in addition to increased OS (Treviño et al., 2015).

On the other hand, there are studies in mice where memory deficits were quickly reversed by switching the animals from an HFD to a low-fat diet (McLean et al., 2018). The above was seen even after prolonged exposure to HFD-feeding (24 weeks), where after returning to a regular diet, the animals did not present learning deficits or spatial memory impairment (Leyh et al., 2021), suggesting that these impairments might be reversible, at least at some point.

A relationship between abdominal adiposity and cognitive decline has been reported regarding human studies where cognitive behavior was analyzed in obese patients. A negative association between anthropometric measurements, such as BMI and waist circumference, and the detriment in some cognitive tasks was proposed (Dye et al., 2017). Nevertheless, Ntlholang et al. (2018) found that in older adults, central adiposity was a stronger predictor of poor cognitive performance than BMI in older adults. In that study, the neuropsychological assessment determinations included the Mini-Mental State Examination (MMSE), Frontal Assessment Battery (FAB), and Repeatable Battery for the Assessment of Neuropsychological Status (RBANS). This was confirmed by Gardener et al. (2021) where a neuropsychological battery evaluating cognitive domains (episodic memory, processing speed, semantic memory, and executive function) was evaluated in obese patients over 65 years of age, and a detrimental effect of mid-life rather than later life was found. Interestingly, abdominal adiposity was an important factor related to cognitive impairment and decline; however, overall adiposity (determined as BMI) was not a risk factor. Something similar was reported by Morys et al. (2021), where obesity was associated with cognitive impairment cerebrovascular disease.

Moreover, magnetic resonance imaging (MRI) has been used to assess neuroinflammation and axonal integrity to determine if there are similar effects in obese humans as observed in rodents. The results show increased cell density related to neuroinflammation and decreased axonal density in obese humans positively correlating with BMI, but not with age (Kullmann et al., 2020; Samara et al., 2020).

These studies support the relationship between obesity and cognitive health and attract the researchers’ attention because obesity may have an immediate and long-term detrimental impact on cognitive functions. The problem is that the molecular mechanisms that participate in obesity-related cognitive decline are diverse, including OS, metabolic dysfunction, cardiovascular disease, and systemic inflammation (Ajayi et al., 2021), highlighting those that are related to the impairment of vascular components where the integrity of the BBB is lost and the microglia are activated (Buie et al., 2019). Therefore, it is of paramount importance to continue researching the mechanisms of action and the interventions to reverse obesity’s detrimental effects. Mainly because in both, humans and rodents, the effectiveness of weight loss (through restrictive diets or other procedures) has been observed to rescue some aspects of neuroinflammation and defects in cognition and behavior (Guillemot-Legris and Muccioli, 2017).

### Differential Inflammatory Response Between Sexes During Obesity

There is a differential response in adiposity and the prevalence of obesity-associated diseases between males and females, particularly in mammals. In obese men, the occurrence of heart disease and myocardial infarction is higher, while in women, obesity is associated with ischemic stroke (Chen et al., 2021). When comparing men and women with the same BMI, it has been observed that women have a 10% higher body fat content. In addition, women show a greater subcutaneous fat volume than men, while men have a larger volume of intra-abdominal or visceral fat (Griffin et al., 2016).

Still, there are limited data to explain the origin of these differences, but several studies have proposed estrogen’s role in the sex-dependent differential responses in metabolism. The decrease in estrogen levels in menopausal women is associated with the loss of subcutaneous fat and the abdominal fat accumulation (Lizcano and Guzmán, 2014). In support of estrogen importance, increased adiposity after oophorectomy and ovarian estrogen clearance were observed in rodents and monkeys (Chen et al., 2021). In the case of men, low testosterone levels have also been proposed as a risk factor for pathophysiology, including insulin sensitivity and DM2 (Terrazas et al., 2019).

On the other hand, the association between sex and OS is significant because OS is involved in many diseases that occur differentially in men and women (Kander et al., 2017). Previous studies revealed that oxidative and nitrosative stress markers are higher in obese men compared to obese women of the same age. Although it is difficult to determine whether these interactions are additive or synergistic, most redox biomarkers depend not only on age and sex but also on age-sex or age-obesity interactions (Choromańska et al., 2021).

Recently, several reports comparing the cognitive impairment by sex associated with obesity have been published. Wang et al. (2021) linked blood lipid levels and obesity using and index named lipid accumulation product (LAP). They reported that high LAP is associated with cognitive decline in females with normal blood pressure but not in those with high blood pressure or males. Suggesting that there is a relationship between obesity and cognitive decline that is differentially affected by blood pressure and sex. Hu et al. (2021) informed that in an older Chinese population, BMI and hip circumference are positively related to cognitive function in women, while no association was found in men. Conversely, another study was performed by Espeland et al. (2021) where older women and men (mean age 68 years old) with DM2 and overweight or obesity were evaluated. Cognitive advantages for women with DM2 and overweight/obesity over men during aging were observed. These differences given by sex are yet uncertain, but their understanding is important since the therapeutic targets and treatments may present variations and should be specifically directed toward men or women.

## Interventions to Reduce the Effects of Obesity

### Pharmacological Treatments

Most weight control medications act in the brain to stimulate satiety signals, motivationally helping the patients adhere to their dietary interventions, with the primary goal of weight loss. Medical guidelines recommend seven drug treatments for weight control, including orlistat, liraglutide, phentermine, phentermine/topiramate, lorcaserin, and naltrexone/bupropion (Lei et al., 2021).

Phentermine/topiramate therapy is known to significantly decrease body weight compared to placebo, and the amount of weight loss has been related to the used dose. Another beneficial effect of phentermine/topiramate treatment was the waist-circumference reduction, blood pressure, blood sugar levels, and lipid levels decrease. However, this drug combination risks adverse events related to the nervous system (Lei et al., 2021).

Orlistat has been well studied in different obese populations, including DM2 and patients with impaired glucose tolerance. Overall, a modest but significant weight loss was observed in all the groups with favorable effects on obesity comorbidities. Orlistat has not been associated with severe adverse events and only mild gastrointestinal effects have been reported in some patients. In obese patients who do not have diabetes, weight loss is achieved and maintained for 2 years. Orlistat, together with a hypocaloric diet, was shown to be effective in preventing DM2 in patients with glucose intolerance and significantly lowers glycated hemoglobin levels (Hollander, 2003).

The possible benefits of using liraglutide for long periods of time have been investigated in people with a BMI greater than 30 or 27 kg/m^2^ associated with dyslipidemia or hypertension. Subjects treated with liraglutide achieved significant weight loss vs. the placebo group. Moreover, when the drug was combined with physical activity, it significantly increased weight loss compared to liraglutide alone or physical exercise alone. These results reinforce the benefits of liraglutide in weight loss and emphasize the fundamental role of physical activity in chronic weight control (Lundgren et al., 2021; Tilinca et al., 2021).

Treatment with phentermine 37.5 mg/day for 3 months to reduce obesity showed a percentage of total weight loss of 7.65% and a more significant reduction in BMI –3.16 kg/m^2^ compared to Lorcaserin 10 mg/2 times a day with a total weight loss of 2.99% and a BMI reduction of –1.15 kg/m^2^. In this same study, the administration of phentermine was performed in a group of patients who had received bariatric surgery but had regained weight and who were subsequently treated with pharmacotherapy, patients using Lorcaserin had a 1.86% total weight loss vs. at 7.62% for phentermine and a smaller BMI reduction of –0.74 vs. –3.06 kg/m^2^ for phentermine. Lorcaserin treatment showed a significant decrease in total cholesterol and low density lipoprotein (LDL) only among surgical patients with a significant weight reduction (≥5% total weight). Both drugs were not associated with glycemic improvements, and no differences were observed between the surgical and non-surgical groups (Elhag et al., 2019).

Naltrexone/bupropion (NB) has also been used as an interesting combination therapy to treat weight and risk factors related to overweight and obesity. A double-blind, placebo-controlled study with 1496 obese patients with BMI 30–45 kg/m^2^ or overweight 27–45 kg/m^2^ with dyslipidemia and/or hypertension was conducted. A significant weight loss was observed with NB (–6.5%) vs. placebo (–1.9%) at week 28 of treatment, and at week 56 a reduction of –6.4% in NB vs. –1.2% in the placebo. NB enhanced different markers related to cardiometabolic risk, and the participants reported improvement in the quality of life. The most common adverse event with NB was nausea, which was generally mild to moderate and transient. NB was not associated with increased depression events or suicidal tendencies compared to placebo (Apovian et al., 2013).

These drugs have beneficial effects; however, numerous medications have been withdrawn due to potentially dangerous or undesirable side effects. In the face of the adverse side effects of synthetic drugs, natural products have been explored, as they are considered non-toxic and healthy. Different dietary, herbal, and natural products, and their active components have been analyzed for their potential anti-obesity effects (Sun et al., 2016).

### Natural Products Against Obesity

There is a long list of natural compounds that have been used to control obesity. Examples of those molecules are alkaloids (capsaicin, caffeine, nicotine), terpenoids (lycopene, lutein, carotene), phytosterols (diosgenin, guggulsterone), organosulfur compounds (allyl sulfide, allicin, allixin), phenolic acids (ferulic, chlorogenic, and caffeic acids), curcuminoids (curcumin), chalcones (naringenin), lignans (matairesinol), flavonoids (kaempferol, quercetin, catechins, cyanidin), isoflavones (genistein), and stilbenes (resveratrol). Anti-obesity effects of these products include energy expenditure stimulants, appetite suppressants, α-amylase, α-glucosidase, lipase inhibitors, adipocyte differentiation inhibitors (decreased adipogenesis), increased lipolysis, or a combination of these effects (Mohamed et al., 2014; Sun et al., 2016).

In this review, we have discussed that obesity-induced inflammation is considered a potential mechanism that links this disease to neuroinflammation and cognitive decline, so targeting obesity-related inflammatory components is proposed as a valuable strategy to prevent or ameliorate the development of such CNS detrimental effects (Hirai et al., 2010).

#### Effect of Dietary Products on Obesity-Associated Neuroinflammation

*Momordica charantia* (bitter melon) has been reported to reduce brain OS and FoxO, as well as normalize neuroinflammatory markers (NFκB, IL16, IL22, and IL17R) in the brain of female mice fed with HFD (Nerurkar et al., 2011). Green tea extract ameliorates HFD-induced hypothalamic inflammation reducing the increase in TLR4, IκB-α, NF-κB p50, and IL6 in mice (Okuda et al., 2014). Epigallocatechin gallate, the major polyphenol in green tea, inhibited HFD-induced obesity by enhancing BAT thermogenesis and diminishing the hypothalamic inflammation and microglia overactivation through NF-κB and STAT3 pathway regulation (Zhou et al., 2018). In another study, this green tea compound was found to attenuate hypothalamic inflammation inhibiting the JAK2/STAT3 signaling pathway in HFD-induced obese mice (Mao et al., 2019).

Anthocyanin-rich blackberry extract counteracted HFD-induced dysbiosis, and modifications in gut microbiota were linked to its anti-neuroinflammatory effect (Marques et al., 2018). Purple sweet potato anthocyanin pigment diminished neuroinflammation induced by HFD in mice by inhibiting MAPK and NF-κB activation, downregulating the expression of iNOS, COX2, IL1β, IL6, and TNFα, and raising IL10 expression (Li J. et al., 2018). Several studies support the use of dietary anthocyanins coming from fruits, vegetables, and beans against DM2-mediated Alzheimer’s disease (Khan et al., 2021).

Peel extract of pineapple fruit protects against HFD-induced behavioral disturbances by decreasing the risk of atherogenicity due to anti-inflammatory, and antioxidant effects. The extract improves brain antioxidant status by increasing reduced glutathione (GSH) and catalase and decreasing IL6 and malondialdehyde (MDA) levels (Ajayi et al., 2021).

#### Effect of Herbal Products on Obesity-Associated Neuroinflammation

Dry leaf powder of *Withania somnifera* used in ayurvedic formulations ameliorated HFD-induced neuroinflammation, suppressing the expression of inflammatory markers (PPARγ, iNOS, MCP1, TNFα, IL1β, and IL6) (Kaur and Kaur, 2017). Furthermore, Xuefu Zhuyu decoction, a traditional Chinese medicine, reduced insulin and leptin levels, neuroinflammation, astrocyte and microglia activation, and amyloid deposition in an animal model of Alzheimer’s disease (Yeh et al., 2017). In this model, an ethyl acetate extract of leaves of *Ugni molinae* Turcz containing tannins, flavonoid derivatives, phenolic acids, and pentacyclic triterpenoids exhibited neuroprotective, anti-inflammatory, and anti-oxidative properties (Jara-Moreno et al., 2018).

*Malva parviflora* used in traditional medicine in Africa and America has anti-inflammatory, antioxidant, and hypoglycemic effects. In a recent study, the anti-inflammatory effect of a hydroalcoholic leaf extract was found to ameliorate HFD effects in an obese transgenic 5XFAD mouse model of Alzheimer’s disease. This extract, which contains oleanolic and scopoletin as active compounds, suppresses neuroinflammation by inhibiting microglia pro-inflammatory M1 phenotype and rescuing microglia phagocytosis via a PPAR-γ/CD36 dependent mechanism (Medrano-Jiménez et al., 2019).

A *Mucuna pruriens* (L.) extract rich in oligosaccharide (1-kestose and levodopa) and phenolic compounds (catechins, chlorogenic acid, trans-resveratrol, and kaempferol 3-glucoside), reduced food intake, neuroinflammation, and hippocampal IL6 levels of obese rats (Tavares et al., 2020). Moreover, Ghaddar et al. (2020) reported that *Antirhea borbonica* herbal tea prevents BBB leakage, cerebral OS, and partly improves neurogenesis in a diet-induced overweight zebrafish model.

*Tinospora cordifolia* extract supplemented in HDF-fed female rats reduced anxiety-like behavior and improved locomotor behavior by decreasing the expression of inflammatory cytokines, modulating apoptosis, and synaptic plasticity (Singh et al., 2021).

Finally, new approaches such as molecular docking studies targeting microglia-specific proteins support using some natural products (like curcumin, cannabidiol, and resveratrol) as possible candidates to regulate redox imbalance, OS, and neuroinflammation (Maurya et al., 2021).

### Diets and Exercise

The primary strategy for treating obesity is diet supplemented with physical exercise and cognitive-behavioral therapy. Low-calorie diets are the most recommended to start reducing body weight. However, these dietary regimens must be supplemented with macronutrients, vitamins, and minerals. The 2015–2020 Dietary Guideline for Americans recommends that carbohydrates comprise 45–65% of calories, fat 25–35% of calories, and protein 10–30% of calories. Once the desired body weight has been reached, the number of calories consumed in the diet can be gradually increased to balance the calories consumed and calories expended.

Regular physical exercise improves the balance between energy consumed and expended, thereby gradually improving the diet’s effectiveness and maintaining diet-induced weight loss. There is a weight loss of 5–8.5 kg in 6 months after the intervention through calorie restriction and exercise. After 48 months, an average of 3–6 kg of the weight loss was maintained (Fock and Khoo, 2013; Bales and Porter-Starr, 2018). Likewise, combining a hypocaloric diet with supervised aerobic exercise 2 days a week offers an optimal non-pharmacological tool in managing blood pressure, cardiorespiratory conditions, and body composition in overweight/obese and sedentary people with hypertension (Gorostegi-Anduaga et al., 2018).

In regards to neuroinflammation, exercise has also shown very promising results. Exercise on a treadmill reduced the levels of inflammation markers such as TNFα, IL1β, and COX2 in the hippocampus of 8-month-old Sprague–Dawley rats on an HFD diet (Kang et al., 2016). Additionally, exercise decreased the activation of microglia and astrocytes in the cerebral cortex and hippocampus compared to sedentary rats fed with HFD (Koga et al., 2014). In HFD obese mice, treadmill exercise enhanced cognitive function by improving neuroplasticity and brain-derived neurotrophic factor (BDNF) expression (Kim et al., 2016). In another study, voluntary physical activity (wheel running) increased hippocampal neurogenesis and spatial learning in female C57BL/6 mice fed with HFD (Klein et al., 2016). Likewise, C57BL/6J (B6) mice fed with a western diet from 2 to 12 months of age, prevented cerebrovascular and white matter damage by free access to running saucer wheels exercise (Graham et al., 2019).

## Conclusion and Perspectives

As discussed throughout this paper, obesity is a severe health problem associated with many diseases, including neuroinflammation and cognitive decline. To date, multiple interventions have been proposed to minimize or prevent neuroinflammation and cognitive impairment. However, the most important would be to develop prevention programs to teach people to eat healthily and perform an adequate exercise regimen.

## Author Contributions

VS-V and MK collaborated in writing of the manuscript, integrated the information, and revised the final version. RF-T, YR-C, DR-R, RR-C, LC-C, LP-F, and AA-A collaborated in writing of the manuscript. NL-D, BG-G, and AC collaborated in writing of the manuscript and revised the final version. All authors contributed to the article and approved the submitted version.

## Conflict of Interest

The authors declare that the research was conducted in the absence of any commercial or financial relationships that could be construed as a potential conflict of interest.

## Publisher’s Note

All claims expressed in this article are solely those of the authors and do not necessarily represent those of their affiliated organizations, or those of the publisher, the editors and the reviewers. Any product that may be evaluated in this article, or claim that may be made by its manufacturer, is not guaranteed or endorsed by the publisher.
